# Artificial Intelligence in Decision-Making for Colorectal Cancer Treatment Strategy: An Observational Study of Implementing Watson for Oncology in a 250-Case Cohort

**DOI:** 10.3389/fonc.2020.594182

**Published:** 2021-02-04

**Authors:** Batuer Aikemu, Pei Xue, Hiju Hong, Hongtao Jia, Chenxing Wang, Shuchun Li, Ling Huang, Xiaoyi Ding, Huan Zhang, Gang Cai, Aiguo Lu, Li Xie, Hao Li, Minhua Zheng, Jing Sun

**Affiliations:** ^1^ Department of General Surgery, Ruijin Hospital, Shanghai Jiao Tong University School of Medicine, Shanghai, China; ^2^ Shanghai Minimally Invasive Surgery Center, Ruijin Hospital, Shanghai Jiao Tong University School of Medicine, Shanghai, China; ^3^ Department of Radiology, Ruijin Hospital, Shanghai Jiao Tong University School of Medicine, Shanghai, China; ^4^ Department of Radiation Oncology, Ruijin Hospital, Shanghai Jiao Tong University School of Medicine, Shanghai, China; ^5^ Clinical Research Institute, Shanghai Jiao Tong University School of Medicine, Shanghai, China; ^6^ Department of Oncology, Ruijin Hospital, Shanghai Jiao Tong University School of Medicine, Shanghai, China

**Keywords:** Watson for Oncology, artificial intelligence, colorectal cancer, multidisciplinary team, concordance analysis

## Abstract

**Background:**

Personalized and novel evidence-based clinical treatment strategy consulting for colorectal cancer has been available through various artificial intelligence (AI) supporting systems such as Watson for Oncology (WFO) from IBM. However, the potential effects of this supporting tool in cancer care have not been thoroughly explored in real-world studies. This research aims to investigate the concordance between treatment recommendations for colorectal cancer patients made by WFO and a multidisciplinary team (MDT) at a major comprehensive gastrointestinal cancer center.

**Methods:**

In this prospective study, both WFO and the blinded MDT’s treatment recommendations were provided concurrently for enrolled colorectal cancers of stages II to IV between March 2017 and January 2018 at Shanghai Minimally Invasive Surgery Center. Concordance was achieved if the cancer team’s decisions were listed in the “recommended” or “for consideration” classification in WFO. A review was carried out after 100 cases for all non-concordant patients to explain the inconsistency, and corresponding feedback was given to WFO’s database. The concordance of the subsequent cases was analyzed to evaluate both the performance and learning ability of WFO.

**Results:**

Overall, 250 patients met the inclusion criteria and were recruited in the study. Eighty-one were diagnosed with colon cancer and 189 with rectal cancer. The concordances for colon cancer, rectal cancer, or overall were all 91%. The overall rates were 83, 94, and 88% in subgroups of stages II, III, and IV. When categorized by treatment strategy, concordances were 97, 93, 89, 87, and 100% for neoadjuvant, surgery, adjuvant, first line, and second line treatment groups, respectively. After analyzing the main factors causing discordance, relative updates were made in the database accordingly, which led to the concordance curve rising in most groups compared with the initial rates.

**Conclusion:**

Clinical recommendations made by WFO and the cancer team were highly matched for colorectal cancer. Patient age, cancer stage, and the consideration of previous therapy details had a significant influence on concordance. Addressing these perspectives will facilitate the use of the cancer decision-support systems to help oncologists achieve the promise of precision medicine.

## Introduction

Colorectal cancer (CRC) is the third most commonly diagnosed cancer in both men and women worldwide (1). Its incidence and mortality rates have been increasing in China for several decades (2). The rapid expansion of clinical databases and massive genetic profiling programs has raised tremendous challenges for oncologists where there is insufficient time for tracking the treatment-related information (3).

Clinical decision-support systems that have emerged in the early days, called expert systems (4), are computer programs that help clinicians manage the comprehensive demands of relevant information developments. These systems collect and analyze knowledge in ways that allow algorithms to simulate human reasoning to assist decision-making. AI systems in cancer care have generally focused on obtaining information from unstructured data such as text (using natural language processing) or large structured datasets (using machine-learning methods) (5). However, a cognitive-support computer program for cancer treatment has, as far as we know, not emerged until the development of IBM’s Watson for Oncology (WFO).

Despite substantial computer science and clinical expertise, mainly from Memorial-Sloan-Kettering Cancer Centre (MSKCC), guided the development of IBM WFO, which holds promise for improving the value of cancer care delivery, the prospects for its use in patients outside the US have not been examined clearly. According to the reports from oncologists in China and other countries, concordance of treatment decisions made by physicians and WFO varies depending on cancer type, where outcomes in terms of breast cancer (5), lung cancer (6), and gastric cancer (7) were likely to be highly concordant, the results in other studies (8, 9) were not.

Hence, we carried out this prospective study to assess the level of agreement regarding colorectal cancer treatment between WFO and a multidisciplinary cancer team in a major comprehensive gastrointestinal cancer center in Shanghai, China. We report the results of decision concordance using the AI system and performed an in-depth analysis on patients where concordance was absent to update the AI model and discuss the potential value of the technology as a clinical adviser and a learning system in cancer treatment.

## Patients and Methods

### Study Design

**Figure 1:**
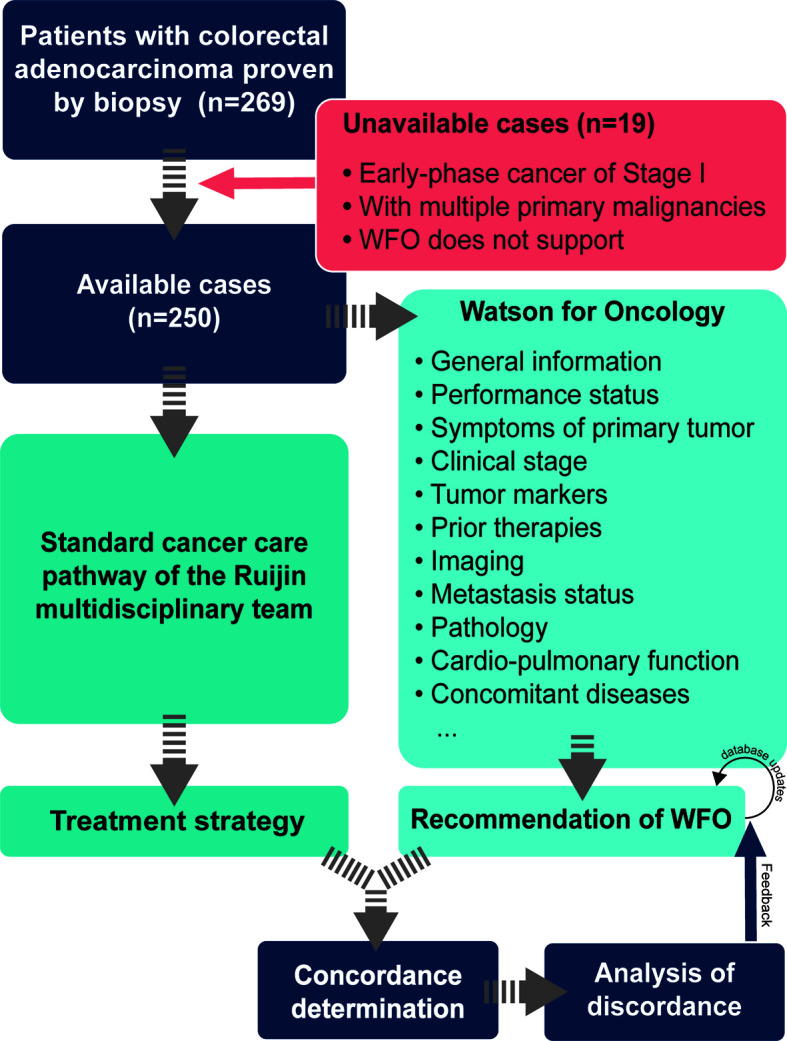
Flow diagram of the study.

This is a prospective, double-blind, and self-controlled trial to evaluate the clinical conformance between WFO and the multidisciplinary team of Ruijin Hospital Affiliated to Shanghai Jiao Tong University School of Medicine (henceforward the RJ MDT) in patients undergoing colorectal cancer therapy in the gastrointestinal center. The clinic information of patients was entered into WFO with patients’ consent, and the results were compared with those of actual clinical treatment plans made by the RJ MDT (**Figure 1**). This study was approved by the ethics committee of Ruijin Hospital.

### Patients

Patients admitted to Ruijin Hospital between March 2017 and January 2018 were eligible for the trial if they were aged between 18 years and 90 years and were diagnosed with colorectal cancer proven by colonoscopy biopsy. All patients provided informed written consent and were advised of their extensive rights to know about related information of the study.

#### Inclusion Criteria

1. Age between 18 and 90 years.
2. Diagnosis of colorectal adenocarcinoma proven by colonoscopic biopsy.
3. Clinical or radiological evidence of Stage II (T3-4, N0, M0), Stage III (T1-4, N1-2, M0), or Stage IV (T1-4, N0-2, M1) disease [according to the Eighth Edition Cancer Staging Manual of the American Joint Committee for Cancer (10)].
4. Could provide all the tumor-related information required by WFO.
5. Patients with recurrence or metastasis should provide the time of recurrence at least.
6. Have signed the informed consent.

#### Exclusion Criteria

1. Multiple primary tumors.
2. Pregnancy.
3. Cases not supported by WFO.
4. Refusal to accept standardized therapy according to the guidelines.

### Procedure

#### Strategy Determined by the RJ MDT Team

The clinical information of patients was analyzed by the RJ MDT, which includes multiple experts from the Departments of General Surgery (including experts specialized in gastrointestinal, hepatobiliary, and pancreatic surgery), Oncology, Radiology, Radiation Oncology, Intervention and Radiotherapy, and Pathology. The treatment plan for each patient was decided according to the guidelines of The National Comprehensive Cancer Network (NCCN), the European Society for Medical Oncology (ESMO), and the Chinese Society of Clinical Oncology (CSCO). Clinical-experience-based treatment suggestion was given when the guidelines recommended various strategies. None of the clinical decisions by RJ MDT was influenced by WFO’s recommendations.

#### Decision Made by WFO

Watson for Oncology (IBM Corporation, USA, version 17.3-17.11) used in our study was provided by Hangzhou Cognitive Network Technology Co., Ltd. (Hangzhou CognitiveCare). The database was updated every 1–2 months by the training team at MSKCC. The patients’ clinical information entered into WFO included general information, performance status, tumor-related symptoms, clinical stage, laboratory examination, prior treatment, imaging, metastasis status, pathology, and other essential data. It generates patient-specific treatment recommendations in three categories: “Recommended” is strongly evidence-supported, “For consideration” is a potentially suitable evidence-based alternative considered by oncologists based on their clinical judgment, and “Not recommended” is treatment with contraindications or strong evidence against its use. If the recommendation involves drug treatment, WFO will mention the therapeutic dose and treatment mode, as well as adverse reactions, risks, and treatment measures for the adverse reactions. If WFO cannot give an accurate judgment, it could recommend global ongoing clinical trials suitable for this case.

#### Comparison

WFO and the doctors in charge of running the system were blinded to the treatment strategies that had been made by the RJ MDT. Concordance was assessed based on how the MDT’s therapy strategy was categorized in WFO’s recommendation list. If MDT’s decision matched the “recommended” or “for consideration” categories, it was designated as concordant. If the decision was either in the “not recommended” table or not listed, the case was defined as non-concordant.

#### Statistics and Analysis

Descriptive statistics of patients’ characteristics were presented using Microsoft Excel. Concordance was presented as percent agreement. Overall survival (OS) was calculated by the Kaplan-Meier method; the difference between survival curves was determined by the log-rank test. The difference was considered statistically significant when P value was less than 0.05. All analyses were conducted with IBM SPSS 22.0 for macOS (IBM, Chicago, USA).

## Results

### Characteristics of Colorectal Cancer Cases

A majority of enrolled colorectal cancer patients were younger than 75 years old (217/250, 86.80%), while there were 14 (17.28%) and 19 (11.25%) patients over the age of 75 years in the colon and rectal cancer groups, respectively. Overall, 69.2% (173/250) were males, and 30.8% (77/250) were females (**Table 1**). In colon and rectal, phase III cases accounted for 48.15% (35/81) and 68.05% (115/169), respectively, while phase IV ranked second and phased II ranked last (**Table 1**). Categorized by final treatment strategy, adjuvant therapy was the most often implemented recommendation in both groups (40.74% in colon cancer and 39.05% in rectal cancer; **Table 1**), followed by surgery (33.33 and 18.93%, respectively; **Table 1**) and first-line treatment (22.22 and 20.71%, respectively; **Table 1**). There was also a relatively small proportion of cases who underwent neoadjuvant and second-line therapy (**Table 1**).

**Table 1:** Baseline characteristics of patients enrolled.

Characteristics****	Cases, n (%)		
	Colon cancer	Rectal cancer	Total
	(n = 81)	(n = 169)	(n = 250)
**Gender**			
**Male**	54 (66.67)	119 (70.41)	173 (69.20)
**Female**	27 (33.33)	50 (29.59)	77 (30.80)
**Age**			
**<75 years**	67(82.72)	150 (88.76)	217 (86.80)
**≥75 years**	14(17.28)	19 (11.25)	33 (13.20)
**Stage**			
**II**	18 (22.22)	18 (10.65)	36 (14.40)
**III**	39 (48.15)	115 (68.05)	154 (61.60)
**IV**	24 (29.63)	36 (21.30)	60 (24.0)
**Treatment strategy**			
**Adjuvant**	33 (40.74)	66 (39.05)	99 (39.60)
**Surgery**	27 (33.33)	32 (18.93)	59 (23.60)
**Neoadjuvant**	0 (0.00)	30 (17.75)	30 (12.00)
**First Line**	18 (22.22)	35 (20.71)	53 (21.20)
**Second Line**	3 (3.70)	6 (3.55)	9 (3.60)

### Concordance of WFO Treatment Recommendations With the RJ MDT’s Opinions

**Figure 2:**
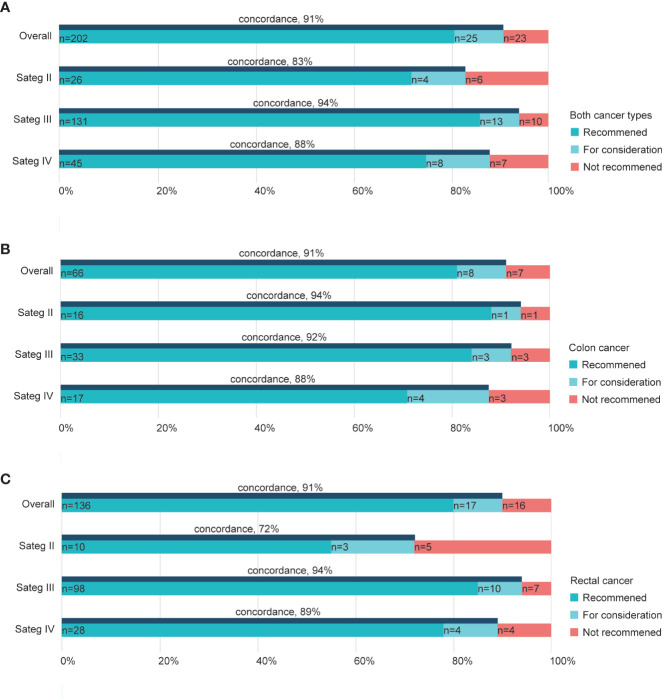
Treatment concordance between WFO and the RJ MDT by stage. Concordance rate in both cancer types **(A)**, colon cancers **(B)**, and rectal cancers **(C)**. RJ MDT, multidisciplinary team of Shanghai Jiao Tong University Medical School affiliated Ruijin Hospital; WFO, Watson for Oncology.

Of the 250 patients treated by the RJ MDT experts and WFO in total, the overall concordance was 91% (**Figure 2A**). Subgroups based on the cancer phase showed concordance rates varied by the staging. Overall cases of Stage III exhibited higher concordance (94%) than stages II (83%) and IV cancers (88%; **Figure 2A**), while cases of stage II colon cancer exhibited higher concordance (94%) than stages III (92%) and IV colon cancers (88%; **Figure 2B**). In contrast, stage II rectal cancer cases showed a relatively lower concordance rate (72%) than stages III (94%) and IV rectal cancers (89%; **Figure 2C**).

**Figure 3:**
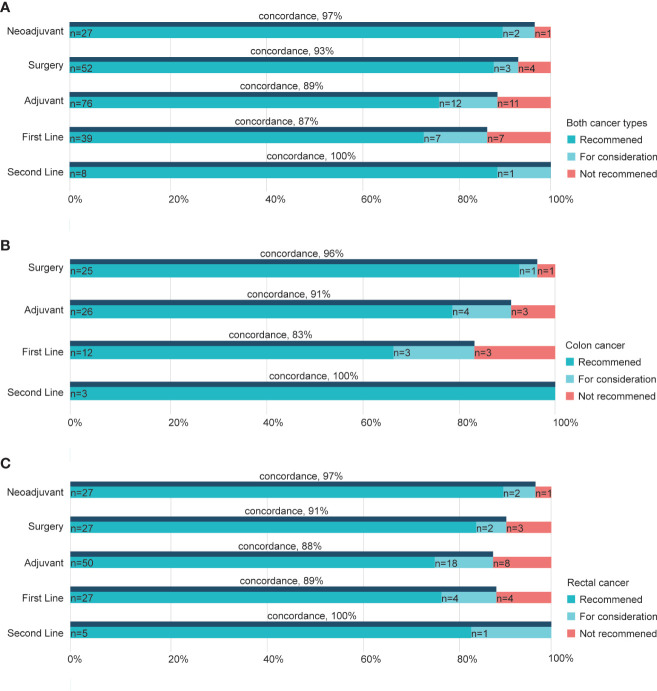
Concordance between WFO and the RJ MDT by treatment strategy. Concordance in subgroups of different strategies in both cancer types **(A)**, colon cancers **(B)**, and rectal cancers **(C)**. RJ MDT, multidisciplinary team of Shanghai Jiao Tong University Medical School affiliated Ruijin Hospital; WFO, Watson for Oncology.

When exploring the concordance based on different treatment strategies, we noticed that the second-line group had the highest concordance rate of 100% for both cancer types (**Figure 3A**). Furthermore, cases recommended undergoing neoadjuvant therapy and surgery had higher concordance (97, 93%, respectively; **Figure 3A**) than the other two, namely adjuvant and first-line groups. Similar results were seen for colon and rectal cancers, where concordance rates were 96 and 91% in surgery groups of colon cancer (**Figure 3B**) and rectal cancer (**Figure 3C**), respectively. Besides, adjuvant therapy for the two cancers showed a 91% concordance rate in colon cancer and 88% in rectal cancer (**Figures 3B, C**). The decisions and recommendations of second-line treatment displayed largely consistent rates of 100% in both cancers (**Figures 3B, C**).

**Figure 4:**
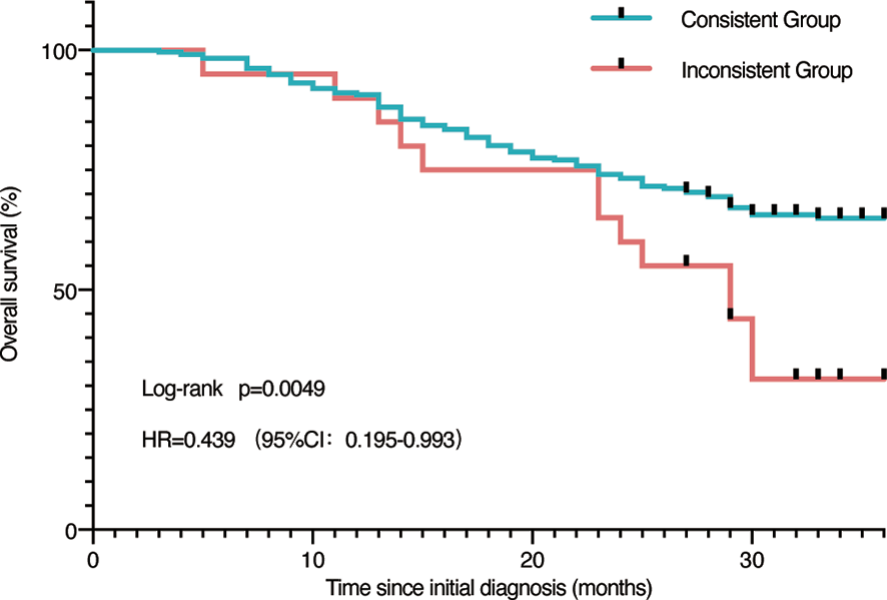
Survival analysis. Survival analysis of all patients grouped by concordance and discordance.

Besides, we speculated if there was a difference in the situation of patients who had consistent or inconsistent results. Patients in the consistent group compared favorably to the inconsistent group (*p* = 0.0049), as shown in **Figure 4**. In the inconsistent group, we observed a median overall survival of 29 months, which was not yet available among the consistent group patients (**Figure 4**).

### Factors Affect the Concordance and Corresponding Updates of WFO

Continuous training was thought to be fundamental to improve the capability of WFO. In applying WFO, we discussed the main reasons resulting in the discordance, and gave feedback to the platform accordingly. We suggested WFO to avoid adjuvant therapy in patients over 80 years in March 2017 and received positive responses from the supporter (**Table 2**). When treating postoperative high-risk stage II colorectal cancers, we found WFO recommended observing strategy, which was against the CSCO (Chinese Society of Clinical Oncology) guidelines. The reason might be the absence of high-risk factors evaluation in dealing with such cases. In spite of a few unresolved proposals, most problems we reported received feedback of update soon after (**Table 2**).

**Table 2:** Feedback given to WFO and the corresponding updated status.

Feedback time****	Suggestion****	Updated time****
2017.3	Apply adjuvant therapy to patient <80 y	2017.3
2017.5	Genetic analysis should be mandatory	2017.5
2017.5	Stage II: High-Risk Evaluation + MSI test should be added	MSI (2017.9) Risk Evaluation (2018.1)
2017.5	Add previous therapy details	2017.5
2017.4	NCCN/ESMO Guidelines should list as the top priority as well; classic publications recommended	Updated every 3 months
2017.5	Indication for neoadjuvant therapy: CRM/EMVI evaluation	2017.9
2017.5	RFA should be considered as a candidate option for mCRC treatment	IBM feedback pending
2017.8	Evaluation of pCR and NED should be added	IBM feedback pending

MSI, Microsatellite instability; NCCN, National Comprehensive Cancer Network; ESMO, European Society for Medical Oncology; CRM, Circumferential resection margin; EMVI, Extramural vascular invasion; pCR, Pathological complete response; NED, No evidence of disease; RFA, Radio-frequency ablation; mCRC, Metastatic colorectal cancer.

**Figure 5:**
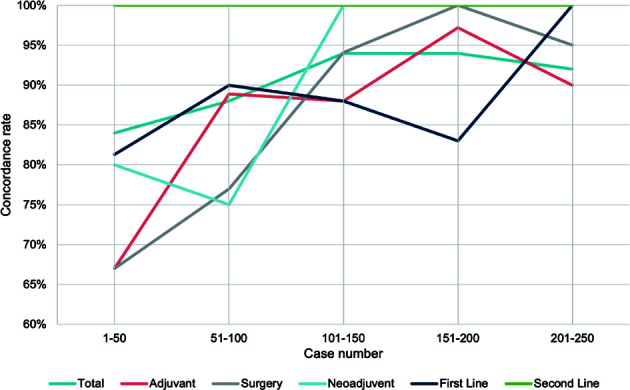
Timeline visualization of the changes in concordance rate. Trend curve of concordance rate in every 50 patients grouped by treatment strategy.

To evaluate the performance of WFO due to its continuous updating database, we analyzed the concordance rate in every 50 cases grouped by treatment strategy. Noticeable rising curves were found in most subgroups of various therapy strategies. Though the concordance met different levels of declines in the last 50 patients in neoadjuvant, surgery, and adjuvant groups, the overall rates were higher than the time applying earlier versions of WFO (**Figure 5**).

## Discussion

The validity and timeliness of clinical guidelines and other therapeutic information an oncologist uses in practice are critical to cancer treatment. With the trends of delegating information-intensive tasks to technologies such as machine learning algorithms, physicians and computer companies are seeking a balance in utilizing evidence-based decision-making support systems in modern clinical practice. While some physicians applied them as a powerful resource, others, especially patients, believed the recommendations they made were already equal to those of the experts. This reflected not only the perspectives and expectations of patients regarding these tools but, more importantly, indicated the concerns of oncologists regarding the validity of the AI-made options. It has been a long time since we introduced such decision-making support systems in real life (4), and the exploration of the most proper model has never ceased.

For such purposes, by examining the concordance between the advice made by WFO, a decision support tool to provide personalized medical recommendations, and an experienced multidisciplinary cancer team, we observed broad agreement and realized the unfulfilled potential of the self-learning machine, as prior studies (11, 12) have suggested. Nevertheless, as we expected, several aspects need to improve. In the early cases, we observed inconsistency in WFO’s recommendations with respect to guidelines. As the classical chemotherapy regimen, FOLFIRI was no longer recommended for adjuvant therapy for stage II or III patients unless enrolled in trials in either the Chinese Society of Clinical Oncology (CSCO) or the National Comprehensive Cancer Network (NCCN) guidelines (13), WFO still listed irinotecan in treatment options. This situation was resolved in the later updated version of WFO. Factors resulting in non-concordance could also come from variations in the aggressiveness of treatment approaches in patient subpopulations based on age. We found in our trial that patients over 80, who were not recommended for aggressive strategies such as chemotherapy in our clinical practice, were likely to have discordance where WFO still recommended standard systemic therapy for this subpopulation. However, the health status of the patients at this age should be rigorously evaluated to manage the benefits and risks of chemotherapy.

Our study also demonstrated that inconsistency between WFO and the RJ MDT occurred in 9% of cases, where the main difference was deriving due to the availability of treatments in China that were not included in the oncology advisor.

China has the largest cancer population with a particular cancer spectrum. The different local conditions and customs of national medicine form different therapeutic experiences and considerations. Since WFO was NCCN guidelines-based and MSKCC experience-trained AI, inevitable deviation from therapeutic guidelines arose. We suggest that, in the process of localizing WFO or developing similar prospective products in China or places outside the US, it is necessary to take more diverse patients treated in varying care settings into consideration (14). In terms of the poor survival rate of patients with inconsistent results, the worse and more complex status of disease and older age probably have played a crucial role in causing the difference. But it also indicates a potential possibility that the AI-powered supporting system could be used as a clinical assistant to help make decisions with better outcome.

Despite the endless arguments towards the responsibility in AI-assisted clinical decision-making systems (15), the great potentials of computerized decision support tools have been demonstrated in medical practice, and many modern technologies are expanding into this area. Google has developed a deep learning machine that can detect diabetic retinopathy and diabetic macular edema (16). Microsoft is exploiting new technology for automated analysis of radiological images (17). The current and potential AI applications cover not only clinical practice, such as diagnosis, robotic surgery, and translational research, such as drug discovery and repurposing, but also several basic biomedical research fields, including gene function annotation and automated experiments (18).

Multi-gene panel testing has been taken into consideration for prognostic cancer staging in conjunction with the American Joint Committee for Cancer (AJCC) staging (10). By combining genomic factors with conventional TNM staging, some anatomically classified groups (such as T_2_N_0_M_0_, stage 2A) were down- or upgraded and were determined to be more suitable therapy in clinical practice. Because of the trends towards relying more on molecular characteristics, supplementary decision support might be needed (19). *KRAS*, which was involved in NCCN guidelines for colorectal cancer in 2008 for the first time, has proven to be a key biomarker in applying EGFR-targeted therapies. Though *KRAS* and *BRAF* mutations were considered optional considerations of WFO, the decision it made did not always match standard treatment well. In our study, metastatic rectal cancer cases with *RAS* wt were treated with cetuximab according to NCCN guidelines (Version 17.3), and this was absent in WFO’s options. This may be due to the different treatment strategy of Memorial Sloan Kettering Cancer Center, where WFO has been trained.

Additionally, the evolving feature of the clinical value of genetic assays may cause an unprecedented condition in which a given mutation may not lead to actionable events at the time of initial diagnosis but may later become considerable as research progresses become available (20). Therefore, tracking cancer’s somatic mutations and reanalyzing them in an updated data pool would seem to be a potential ability of AI-based technology such as WFO to achieve precision medicine.

Patient perspectives are integral for the advanced use of WFO in the clinical workflow. Though modern societies, especially those in China, hold optimistic views of applying cutting edge technology in life, it raises a concern regarding health care, involving both data security and decision precision. Therefore, achieving higher levels of patient acceptance of WFO through systematically upgrade will not only improve oncology practice but contribute to enhance the relationship of cancer patients and physicians as well. Given that WFO is not yet commonly used in practice at the hospital, future studies should exploit their findings with physicians, as well as patients, in using WFO in clinical practice.

There are notable limitations to this study. First, the study design was observational and self-controlled with a relatively small sample size that may cause the results potentially to be susceptible to the bias of unmeasured factors. Patients participated in our study were treated at one comprehensive gastrointestinal cancer center on China’s east coast. Adding cases treated in community-based clinics might widen the gap between WFO and clinician responses and lower the concordance but improve the value of computer-aided decision support in minimizing the medical disparities across different regions.

Many who were glad to accept WFO as a resource to provide oncologists with cutting-edge medical research and knowledge believed the ideal model of such tools in clinical practice is to be used as “a tool, not a crutch” (21). By addressing such perspectives, we wish to facilitate the use of WFO and other decision support tools, to help realize the promise of more effective clinical and precision healthcare.

## Data Availability Statement

The original contributions presented in the study are included in the article/supplementary material. Further inquiries can be directed to the corresponding authors.

## Ethics Statement

The studies involving human participants were reviewed and approved by Ruijin Hospital Ethics Committee. The patients/participants provided their written informed consent to participate in this study.

## Author Contributions

BA: Investigation, methodology, software, writing—original draft, and editing. PX: Resources, data curation, formal analysis, validation, investigation, and writing—original draft. HH: Resources, formal analysis, methodology, and editing. HJ: Resources, formal analysis, methodology, and editing. CW: Resources, software, formal analysis, and editing. SL: Resources and methodology. LH: Resources, formal analysis, and methodology. XD: Resources. HZ: Resources. GC: Resources. AL: Resources. LX: Resources, methodology, and formal analysis. MZ: Conceptualization, data curation, supervision, acquisition, validation, methodology, writing—original draft, writing—review and editing. HL: Conceptualization, data curation, supervision, acquisition, validation, methodology, writing—original draft, writing—review and editing. JS: Conceptualization, data curation, formal analysis, supervision, acquisition, validation, methodology, investigation, writing—original draft, writing—review and editing. All authors contributed to the article and approved the submitted version.

## Conflict of Interest

The authors declare that the research was conducted in the absence of any commercial or financial relationships that could be construed as a potential conflict of interest.
